# Preoperative glucose-to-lymphocyte ratio predicts survival in cancer

**DOI:** 10.3389/fendo.2024.1284152

**Published:** 2024-03-04

**Authors:** Le Liu, Bei-bei Zhang, Yuan-zhou Li, Wen-juan Huang, Ye Niu, Qing-chun Jia, Wen Wang, Jia-rui Yuan, Shi-di Miao, Rui-tao Wang, Guang-yu Wang

**Affiliations:** ^1^ Department of Internal Medicine, Harbin Medical University Cancer Hospital, Harbin Medical University, Harbin, Heilongjiang, China; ^2^ Department of Radiology, Harbin Medical University Cancer Hospital, Harbin Medical University, Harbin, Heilongjiang, China; ^3^ Department of Science and Education, School of Computer Science and Technology, Harbin University of Science and Technology, Harbin, Heilongjiang, China; ^4^ Department of Gastrointestinal Medical Oncology, Harbin Medical University Cancer Hospital, Harbin Medical University, Harbin, Heilongjiang, China

**Keywords:** cancer, survival, prognosis, glucose to lymphocyte ratio, lung cancer

## Abstract

**Background:**

Systemic inflammation and glucose metabolism have been closely related to the survival of cancer patients. Therefore, we aimed to evaluate whether preoperative glucose-to-lymphocyte ratio (GLR) can be used to predict the survival of cancer patients.

**Methods:**

We retrospectively examined 2172 cancer patients who underwent surgery from January 1, 2014, to December 31, 2016. There were 240 patients with non-small cell lung cancer (NSCLC), 378 patients with colorectal cancer (CRC), 221 patients with breast cancer (BC), 335 patients with gastric cancer (GC), 270 patients with liver cancer, 233 patients with esophageal cancer (EC), 295 patients with renal cancer, and 200 patients with melanoma. The formula for preoperative GLR calculation was as follows: GLR=glucose/lymphocyte count. The overall survival (OS) was estimated using the Kaplan-Meier method. The predictive factors for OS were determined using multivariate analysis.

**Results:**

The Kaplan-Meier analysis showed that the median survival time in the high-GLR group was much shorter than that of those in the low-GLR group for different cancers. Cox multivariate regression analysis reveals that preoperative GLR was an independent factor for predicting overall survival in different tumor types.

**Conclusion:**

Elevated preoperative GLR was remarkably associated with a poorer prognosis in patients with NSCLC, CRC, breast cancer, gastric cancer, kidney cancer, liver cancer, esophageal cancer, and melanoma. Preoperative GLR promises to be an essential predictor of survival for cancer patients.

## Introduction

As the morbidity rate continues to climb, cancer is not only a major public health problem but also one of the leading contributors to death in the world (1). Up to date, surgery resection is still the mainstay of curative treatment options for most tumors (2). However, despite efforts to develop new surgical strategies, overall survival is still unsatisfactory. Therefore, a more accurate evaluation index to predict the long-term survival of patients has high clinical value.

Diabetes mellitus (DM) and cancer are two prevalent disorders that coexist, and the incidence and prevalence of both are rising (3). DM and hyperglycemia have been demonstrated to have significant impacts on the incidence and prognosis of cancer (4–6). Moreover, the metabolic abnormalities in hyperglycemia and diabetes substantially contribute to the development and progression of cancer (7). A meta-analysis revealed that metformin is an independent protective factor for cancer risk in DM patients (8). In addition, large bodies of accumulated research have also confirmed that the development and progression of cancer increase the risk of diabetes (9).

At the same time, lymphocytes, being one of the crucial components of the systemic inflammatory response, are engaged in cell-mediated antitumor responses (10). Furthermore, its profound role in immune surveillance that protects the host from tumor development has also been observed in mice and humans (11).

The available literature demonstrated the potential association of glucose-to-lymphocyte ratio (GLR) with prognosis in gallbladder, colorectal cancer (CRC), and pancreatic cancer (12). However, there are relatively few studies on the prognostic association of GLR with other tumors. The objective of our study is to evaluate the prognostic role of preoperative GLR in patients with gastric cancer (GC), renal carcinoma, colorectal cancer, non-small cell lung cancer, breast cancer (BC), liver cancer, esophageal cancer (EC), and melanoma.

## Patients and methods

### Study population

We reviewed the clinical information of 2172 cancer patients who underwent curative resection at the Harbin Medical University Cancer Hospital between January 1, 2014, and December 31, 2016. There were 240 patients with non-small cell lung cancer, 378 patients with colorectal cancer, 221 patients with breast cancer, 335 patients with gastric cancer, 270 patients with liver cancer, 233 patients with esophageal cancer, 295 patients with renal cell cancer, and 200 patients with melanoma. Patients were included according to the following criteria: (1) pathologically confirmed evidence of each cancer, (2) completed preoperative blood tests involving fasting glucose and lymphocyte counts, and (3) followed for more than 60 months. Exclusion criteria for patients were as follows: (1) they had received antitumor therapy before surgery; (2) they had a history of other primary malignancies; (3) they had acute inflammatory disease; and (4) they failed to follow up.

Overall survival (OS) was defined from the date of surgery to the date of death or the date of the last follow-up. All patients were followed up by telephone once every 3-6 months. The cut-off date for follow-up evaluations is December 31, 2021. The survival data was derived from medical records and telephone follow-ups. And the work has been reported in line with the REMARK criteria (13). Patients’ demographic characteristics and laboratory parameters were extracted from their electronic medical records. All laboratory parameters were assayed within a week before the operation. The formula for preoperative GLR calculation was as follows: GLR=fasting blood glucose (mmol/L)/lymphocyte count (×10^9^/L).

This research was in strict compliance with the Helsinki Declaration. This study was approved by our Institutional Review Board (approval number KY2022-10). Since it was a retrospective study, we waived informed consent.

### Statistical analysis

Statistical tests were performed with SPSS version 25.0 software (SPSS Inc., Chicago, IL, USA). Receiver operating characteristic (ROC) curves were constructed using MedCalc version 15.0 software to assign cut-off values for GLR levels as well as sensitivities and specificities. The Kolmogorov-Smirnov test was used to determine if the data were normally distributed. T-tests were utilized for the comparison of normally distributed continuous variables, while categorical variables were compared with chi-square tests. The Kaplan-Meier method was used to derive OS, and the results were compared with the log-rank test. Multivariate analysis was conducted using the Cox proportional hazards regression model to estimate the independent predictors of OS. The proportional-hazards assumption was examined before Cox regression analysis. Univariate and multivariate Cox regression analyses were carried out to determine the hazard ratio (HR) and the corresponding 95% confidence interval (CI). Variables with a p value of < 0.10 in the univariate analysis were subjected to multivariate analysis. All reported p values were two-sided, and p values < 0.05 were regarded as statistically significant.

## Results

Among the 2172 patients collected, the mean age was 55.72 years (range 10-87), 1265 (58.2%) were men, and 907 (41.8%) were women.

The patient’s clinical characteristics based on preoperative GLR levels are summarized in **Table 1**. In gastric cancer, colorectal cancer, liver cancer, esophageal cancer, and renal cancer, lower hemoglobin and platelet count were likely to appear in the high-GLR group. In non-small cell lung cancer, colorectal cancer, and renal cancer, age in the two groups showed a significant difference. Moreover, high GLR levels were correlated with white blood cell in melanoma, breast cancer, liver cancer, esophageal cancer, renal cancer, and non-small cell lung cancer.

**Table 1:** Patient characteristics according to glucose-to-lymphocyte ratio status.

Variables	Total n (%)	Low GLR	High GLR	P value
Non-small-cell Lung Cancer				
Age (years)				0.022
≤ 60	159 (66.3)	119 (70.8)	40 (55.6)	
> 60	81 (33.8)	49 (29.2)	32 (44.4)	
Gender				0.502
Female	89 (37.1)	60 (35.7)	29 (40.3)	
Male	151 (62.9)	108 (64.3)	43 (59.7)	
Tumor size (cm)				0.586
< 4	164 (68.3)	113 (67.3)	51 (70.8)	
≥ 4	76 (31.7)	55 (32.7)	21 (29.2)	
Smoking history				0.308
No	132 (55.0)	96 (57.1)	36 (50.0)	
Yes	108 (45.0)	72 (42.9)	36 (50.0)	
Hypertension				0.374
No	198 (82.5)	141 (83.9)	57 (79.2)	
Yes	42 (17.5)	27 (16.1)	15 (20.8)	
Diabetes mellitus				0.111
No	223 (92.9)	159 (94.6)	64 (88.9)	
Yes	17 (7.1)	9 (5.4)	8 (11.1)	
Adjuvant chemotherapy				0.775
No	100 (41.7)	71 (42.3)	29 (40.3)	
Yes	140 (58.3)	97 (57.7)	43 (59.7)	
Histology				0.734
Adenocarcinoma	134 (55.8)	95 (56.5)	39 (54.2)	
Others	106 (44.2)	73 (43.5)	33 (45.8)	
T classification				0.154
T1/T2	29 (12.1)	17 (10.1)	12 (16.7)	
T3/T4	211 (87.9)	151 (89.9)	60 (83.3)	
Lymph node status				0.421
Absent	159 (66.3)	114 (67.9)	45 (62.5)	
Present	81 (33.8)	54 (32.1)	27 (37.5)	
Clinical stage				0.126
I/II	176 (73.3)	128 (76.2)	48 (66.7)	
III	64 (26.7)	40 (23.8)	24 (33.3)	
BMI (kg/m^2^)	23.28 ± 3.06	23.31 ± 3.10	23.22 ± 3.20	0.838
WBC (×10^9^/L)	6.92 ± 2.41	7.12 ± 2.36	6.45 ± 2.48	0.048
Hemoglobin (g/L)	137.75 ± 18.24	139.13 ± 18.70	134.51 ± 16.82	0.072
Platelet count (×10^9^/L)	243.95 ± 70.69	243.32 ± 67.23	245.42 ± 78.66	0.834
Colorectal Cancer				
Age (years)				0.002
≤ 65	305 (80.7)	285 (82.6)	20 (60.6)	
> 65	73 (19.3)	60 (17.4)	13 (39.4)	
Gender				0.079
Female	133 (35.2)	126 (36.5)	7 (21.2)	
Male	245 (64.8)	219 (63.5)	26 (78.8)	
Hypertension				0.853
No	280 (74.1)	256 (74.2)	24 (72.7)	
Yes	98 (25.9)	89 (25.8)	9 (27.3)	
Diabetes mellitus				< 0.001
No	328 (86.8)	309 (89.6)	19 (57.6)	
Yes	50 (13.2)	36 (10.4)	14 (42.4)	
T classification				0.248
T1/T2	68 (18.0)	65 (18.8)	3 (9.1)	
T3/T4	310 (82.0)	280 (81.2)	30 (90.9)	
Lymph node status				0.073
Absent	205 (54.2)	192 (55.7)	13 (39.4)	
Present	173 (45.8)	153 (44.3)	20 (60.6)	
Clinical stage				0.108
I/II	188 (49.7)	176 (51.0)	12 (36.4)	
III/IV	190 (50.3)	169 (49.0)	21 (63.6)	
BMI (kg/m^2^)	23.20 ± 3.13	23.34 ± 3.13	21.81 ± 2.75	0.007
WBC (×10^9^/L)	6.47 ± 2.24	6.44 ± 2.17	6.77 ± 2.96	0.425
Hemoglobin (g/L)	129.69 ± 24.43	130.17 ± 23.73	124.66 ± 30.81	0.324
Platelet count (×10^9^/L)	271.62 ± 94.76	275.05 ± 95.10	235.79 ± 84.36	0.023
Breast Cancer				
Age (years)				0.586
≤ 50	112 (50.7)	69 (49.3)	43 (53.1)	
> 50	109 (49.3)	71 (50.7)	38 (46.9)	
Tumor size (cm)				0.727
< 2.5	172 (77.8)	110 (78.6)	62 (76.5)	
≥ 2.5	49 (22.2)	30 (21.4)	19 (23.5)	
Menopausal status				0.882
Pre	86 (38.9)	55 (39.3)	31 (38.3)	
Post	135 (61.1)	85 (60.7)	50 (61.7)	
Hypertension				0.999
No	191 (86.4)	121 (86.4)	70 (86.4)	
Yes	30 (13.6)	19 (13.6)	11 (13.6)	
Diabetes mellitus				0.002
No	214 (96.8)	140 (100.0)	74 (91.4)	
Yes	7 (3.2)	0 (0.0)	7 (8.6)	
ER				0.569
Negative	79 (35.7)	52 (37.1)	27 (33.3)	
Positive	142 (64.3)	88 (62.9)	54 (66.7)	
PR				0.285
Negative	81 (36.7)	55 (39.3)	26 (32.1)	
Positive	140 (63.3)	85 (60.7)	55 (67.9)	
HER2				0.348
Negative	121 (54.8)	80 (57.1)	41 (50.6)	
Positive	100 (45.2)	60 (42.9)	40 (49.4)	
Ki-67				0.196
< 20	135 (61.1)	81 (57.9)	54 (66.7)	
≥ 20	86 (38.9)	59 (42.1)	27 (33.3)	
T classification				0.764
T1/T2	210 (95.0)	134 (95.7)	76 (93.8)	
T3/T4	11 (5.0)	6 (4.3)	5 (6.2)	
Lymph node status				0.559
Absent	184 (83.3)	115 (82.1)	69 (85.2)	
Present	37 (16.7)	25 (17.9)	12 (14.8)	
Clinical stage				0.340
I/II	196 (88.7)	122 (87.1)	74 (91.4)	
III	25 (11.3)	18 (12.9)	7 (8.6)	
BMI (kg/m^2^)	23.69 ± 3.57	23.79 ± 3.75	23.52 ± 3.25	0.583
WBC (×10^9^/L)	6.13 ± 1.71	6.56 ± 1.74	5.38 ± 1.38	< 0.001
Hemoglobin (g/L)	135.36 ± 11.83	135.70 ± 11.72	134.77 ± 12.08	0.577
Platelet count (×10^9^/L)	236.66 ± 50.61	241.16 ± 48.57	228.89 ± 53.39	0.082
Gastric Cancer				
Age (years)				0.906
≤ 65	252 (75.2)	205 (75.1)	47 (75.8)	
> 65	83 (24.8)	68 (24.9)	15 (24.2)	
Gender				0.046
Female	100 (29.9)	75 (27.5)	25 (40.3)	
Male	235 (70.1)	198 (72.5)	37 (59.7)	
Hypertension				0.281
No	276 (82.4)	222(81.3)	54 (87.1)	
Yes	59 (17.6)	51 (18.7)	8 (12.9)	
Diabetes mellitus				0.273
No	313 (93.4)	257 (94.1)	56 (90.3)	
Yes	22 (6.6)	16 (5.9)	6 (9.7)	
Tumor size (cm)				0.386
≤ 5.0	222 (66.3)	178 (65.2)	44 (71.0)	
> 5.0	113 (33.7)	95 (34.8)	18 (29.0)	
Histology				0.022
Well/Moderate	61 (18.2)	56 (20.5)	5 (8.1)	
Poor	274 (81.8)	217 (79.5)	57 (91.9)	
CEA (ng/mL)				0.176
≤ 5 ng/mL	274 (81.8)	227 (83.2)	47 (75.8)	
> 5 ng/mL	61 (18.2)	46 (16.8)	15 (24.2)	
T classification				0.236
T1/T2	83 (24.8)	64 (23.4)	19 (30.6)	
T3/T4	252 (75.2)	209 (76.6)	43 (69.4)	
Lymph node status				0.695
Absent	71 (21.2)	59 (21.6)	12 (19.4)	
Present	264 (78.8)	214 (78.4)	50 (80.6)	
Clinical stage				0.588
I/II	129 (38.5)	107 (39.2)	22 (35.5)	
III/IV	206 (61.5)	166 (60.8)	40 (64.5)	
BMI (kg/m^2^)	22.84 ± 3.51	22.83 ± 3.55	22.89 ± 3.34	0.900
WBC (×10^9^/L)	6.47 ± 2.16	6.57 ± 2.14	6.02 ± 2.19	0.069
Hemoglobin (g/L)	128.28 ± 26.64	129.97 ± 25.41	120.83 ± 30.60	0.032
Platelet count (×10^9^/L)	271.28 ± 94.95	276.40 ± 96.32	248.76 ± 85.76	0.038
Liver Cancer				
Age (years)				0.360
≤ 55	161 (59.6)	119 (61.3)	42 (55.3)	
> 55	109 (40.4)	75 (38.7)	34 (44.7)	
Gender				0.370
Female	141 (52.2)	98 (50.5)	43 (56.6)	
Male	129 (47.8)	96 (49.5)	33 (43.4)	
Hypertension				0.322
No	236 (87.4)	172 (88.7)	644 (84.2)	
Yes	34 (12.6)	22 (11.3)	12 (15.8)	
Diabetes mellitus				1.000
No	270 (100.0)	194 (100.0)	76 (100.0)	
Yes	0 (0.0)	0 (0.0)	0 (0.0)	
Smoking history				0.064
No	172 (63.7)	117 (60.3)	55 (72.4)	
Yes	98 (36.3)	77 (39.7)	21 (27.6)	
Drinking history				0.985
No	224 (83.0)	161 (83.0)	63 (82.9)	
Yes	46 (17.0)	33 (17.0)	13 (17.1)	
Tumor size (cm)				0.722
< 5	129 (47.8)	94 (48.5)	35 (46.1)	
≥ 5	141 (52.2)	100 (51.5)	41 (53.9)	
Hepatitis B				0.662
Absent	58 (21.5)	43 (22.2)	15 (19.7)	
Present	212 (78.5)	151 (77.8)	61 (80.3)	
Liver Cirrhosis				0.002
Absent	100 (37.0)	83 (42.8)	17 (22.4)	
Present	170 (63.0)	111 (57.2)	59 (77.6)	
T classification				0.321
T1/T2	155 (57.4)	115 (59.3)	40 (52.6)	
T3/T4	115 (42.6)	79 (40.7)	36 (47.4)	
Lymph node status				0.971
Absent	252 (93.3)	181 (93.3)	71 (93.4)	
Present	18 (6.7)	13 (6.7)	5 (6.6)	
Clinical stage				0.518
I/II	147 (54.4)	108 (55.7)	39 (51.3)	
III/IV	123 (45.6)	86 (44.3)	37 (48.7)	
BMI (kg/m^2^)	24.00 ± 2.98	23.96 ± 3.11	24.10 ± 2.65	0.724
WBC (×10^9^/L)	5.33 ± 1.85	5.73 ± 1.71	4.33 ± 1.81	< 0.001
Hemoglobin (g/L)	137.47 ± 17.81	139.23 ± 15.74	132.98 ± 21.73	0.024
Platelet count (×10^9^/L)	155.71 ± 73.82	173.75 ± 71.63	109.66 ± 57.88	< 0.001
Esophageal Cancer				
Age (years)				0.667
≤ 65	169 (72.5)	133 (71.9)	36 (75.0)	
> 65	64 (27.5)	52 (28.1)	12 (25.0)	
Gender				0.734
Female	13 (5.6)	10 (5.4)	3 (6.3)	
Male	220 (94.4)	175 (94.6)	45 (93.8)	
Hypertension				0.325
No	201 (86.3)	157 (84.9)	44 (91.7)	
Yes	32 (13.7)	28 (15.1)	4 (8.3)	
Diabetes mellitus				0.177
No	222 (95.3)	174 (94.1)	48 (100.0)	
Yes	11 (4.7)	11 (5.9)	0 (0.0)	
Smoking history				0.152
No	46 (19.7)	33 (17.8)	13 (27.1)	
Yes	187 (80.3)	152 (82.2)	35 (72.9)	
Drinking history				0.854
No	31 (13.3)	25 (13.5)	6 (12.5)	
Yes	202 (86.7)	160 (86.5)	42 (87.5)	
Tumor size (cm)				0.164
< 3.5	40 (17.2)	35 (18.9)	5 (10.4)	
≥ 3.5	193 (82.8)	150 (81.1)	43 (89.6)	
Histology				0.371
Squamous carcinoma	226 (97.0)	178 (96.2)	48 (100.0)	
Others	7 (3.0)	7 (3.8)	0 (0.0)	
T classification				0.050
T1/T2	107 (45.9)	91 (49.2)	16 (33.3)	
T3/T4	126 (54.1)	94 (50.8)	32 (66.7)	
Lymph node status				0.678
Absent	120 (51.5)	94 (50.8)	26 (54.2)	
Present	113 (48.5)	91 (49.2)	22 (45.8)	
Clinical stage				0.132
I/II	100 (42.9)	84 (45.4)	16 (33.3)	
III/IV	133 (57.1)	101 (54.6)	32 (66.7)	
BMI (kg/m^2^)	22.08 ± 2.96	22.06 ± 2.90	22.15 ± 3.19	0.846
WBC (×10^9^/L)	6.90 ± 1.99	7.07 ± 1.82	6.26 ± 2.48	0.039
Hemoglobin (g/L)	142.20 ± 14.09	143.39 ± 13.66	137.60 ± 14.91	0.011
Platelet count (×10^9^/L)	239.18 ± 70.51	241.81 ± 69.95	229.04 ± 72.48	0.265
Renal Cancer				
Age (years)				0.042
≤ 65	235 (79.7)	92 (86.0)	143 (76.1)	
> 65	60 (20.3)	15 (14.0)	45 (23.9)	
Gender				0.278
Female	104 (35.3)	42 (39.3)	62 (33.0)	
Male	191 (64.7)	65 (60.7)	126 (67.0)	
Hypertension				0.037
No	244 (82.7)	95 (88.8)	149 (79.3)	
Yes	51 (17.3)	12 (11.2)	39 (20.7)	
Diabetes mellitus				0.004
No	266 (90.2)	104 (97.2)	162 (86.2)	
Yes	29 (9.8)	3 (2.8)	26 (13.8)	
Smoking history				0.472
No	259 (87.8)	92 (86.0)	167 (88.8)	
Yes	36 (12.2)	15 (14.0)	21 (11.2)	
Drinking history				0.087
No	279 (94.6)	98 (91.6)	181 (96.3)	
Yes	16 (5.4)	9 (8.4)	7 (3.7)	
Tumor size (cm)				0.132
≤ 4.0	121 (41.0)	50 (46.7)	71 (37.8)	
> 4.0	174(59.0)	57 (53.3)	117 (62.2)	
Histology				0.612
Others	27 (9.2)	11 (10.3)	16 (8.5)	
Clear cell	268 (90.8)	96 (89.7)	172 (91.5)	
T classification				0.278
T1/T2	275 (93.2)	102 (95.3)	173 (92.0)	
T3/T4	20 (6.8)	5 (4.7)	15 (8.0)	
Lymph node status				0.451
Absent	285 (96.6)	105 (98.1)	180 (95.7)	
Present	10 (3.4)	2 (1.9)	8 (4.3)	
Clinical stage				0.111
I/II	255 (86.4)	97 (90.7)	158 (84.0)	
III/IV	40 (13.6)	10 (9.3)	30 (16.0)	
BMI (kg/m^2^)	24.21 ± 3.67	24.00 ± 2.85	24.33 ± 4.07	0.450
WBC (×10^9^/L)	6.49 ± 2.03	6.96 ± 1.34	6.22 ± 2.30	0.001
Hemoglobin (g/L)	133.59 ± 20.00	134.60 ± 20.05	133.02 ± 20.00	0.515
Platelet count (×10^9^/L)	242.44 ± 91.19	256.74 ± 90.73	234.30 ± 90.69	0.042
Melanoma				
Age (years)				0.209
≤ 60	126 (63.0)	97 (65.5)	29 (55.8)	
> 60	74 (37.0)	51 (34.5)	23 (44.2)	
Gender				0.250
Female	94 (47.0)	66 (44.6)	28 (53.8)	
Male	106 (53.0)	82 (55.4)	24 (46.2)	
Hypertension				0.643
No	161 (80.5)	118 (79.7)	43 (82.7)	
Yes	39 (19.5)	30 (20.3)	9 (17.3)	
Diabetes mellitus				0.713
No	185 (92.5)	138 (93.2)	47 (90.4)	
Yes	15 (7.5)	10 (6.8)	5 (9.6)	
Tumor location				0.361
Sun-exposed (head and neck)	20 (10.0)	17 (11.5)	3 (5.8)	
Sun-protected (others)	180 (90.0)	131 (88.5)	49 (94.2)	
Ulceration				0.617
Negative	125 (62.5)	91 (61.5)	34 (65.4)	
Positive	75 (37.5)	57 (38.5)	18 (34.6)	
Histology				0.201
SSM/NM	104 (52.0)	73 (49.3)	31 (59.6)	
ALM/LMM/others	96 (48.0)	75 (50.7)	21 (40.4)	
T classification				0.293
T1/T2	167 (83.5)	126 (85.1)	41 (78.8)	
T3/T4	33 (16.5)	22 (14.9)	11 (21.2)	
Lymph node status				0.656
Absent	147 (73.5)	110 (74.3)	37 (71.2)	
Present	53 (26.5)	38 (25.7)	15 (28.8)	
Clinical stage				0.315
I/II	138 (69.0)	105 (70.9)	33 (63.5)	
III/IV	62 (31.0)	43 (29.1)	19 (36.5)	
BMI (kg/m^2^)	24.42 ± 3.41	24.46 ± 3.51	24.29 ± 3.14	0.757
WBC (×10^9^/L)	6.25 ± 2.06	6.47 ± 1.89	5.63 ± 2.41	0.011
Hemoglobin (g/L)	138.49 ± 27.72	139.24 ± 30.21	136.36 ± 19.01	0.520
Platelet count (×10^9^/L)	234.63 ± 66.98	237.76 ± 61.10	225.71 ± 81.47	0.266

Supplement: SSM, superficial spreading melanoma; NM, nodular melanoma; ALM, acromacular melanoma; LMM, lentigo maligna melanoma.

**Figure 1:**
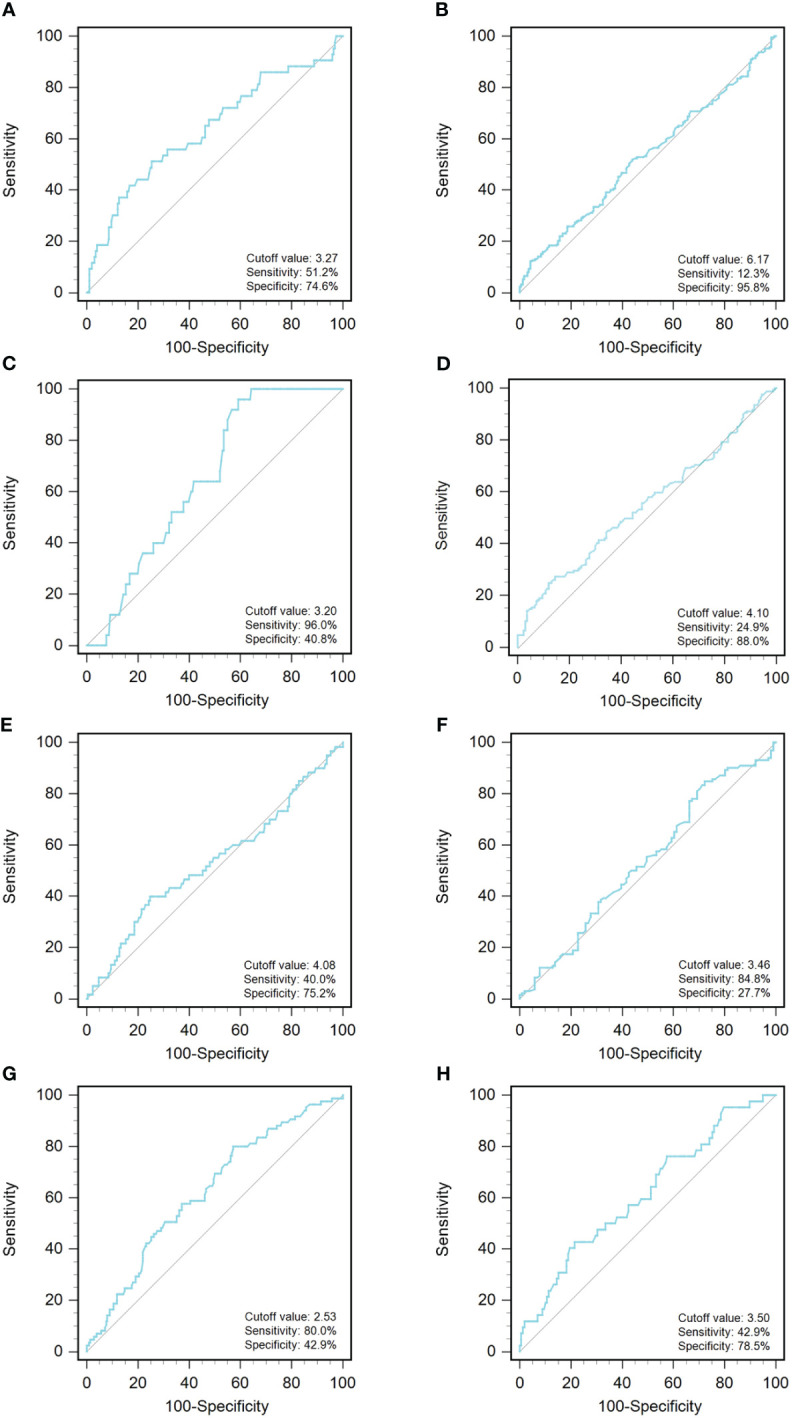
An optimized cut-off value was determined for preoperative GLR using ROC curve analysis. The ROC curve identified the optimal cutoff value of GLR with sensitivity and specificity. **(A)** non-small-cell lung cancer; **(B)** colorectal cancer; **(C)** breast cancer; **(D)** gastric cancer; **(E)** liver cancer; **(F)** esophageal cancer; **(G)** renal cancer; and **(H)** melanoma. ROC curve, receiver operating characteristic curve; GLR, glucose to lymphocyte ratio.

**Figure 2:**
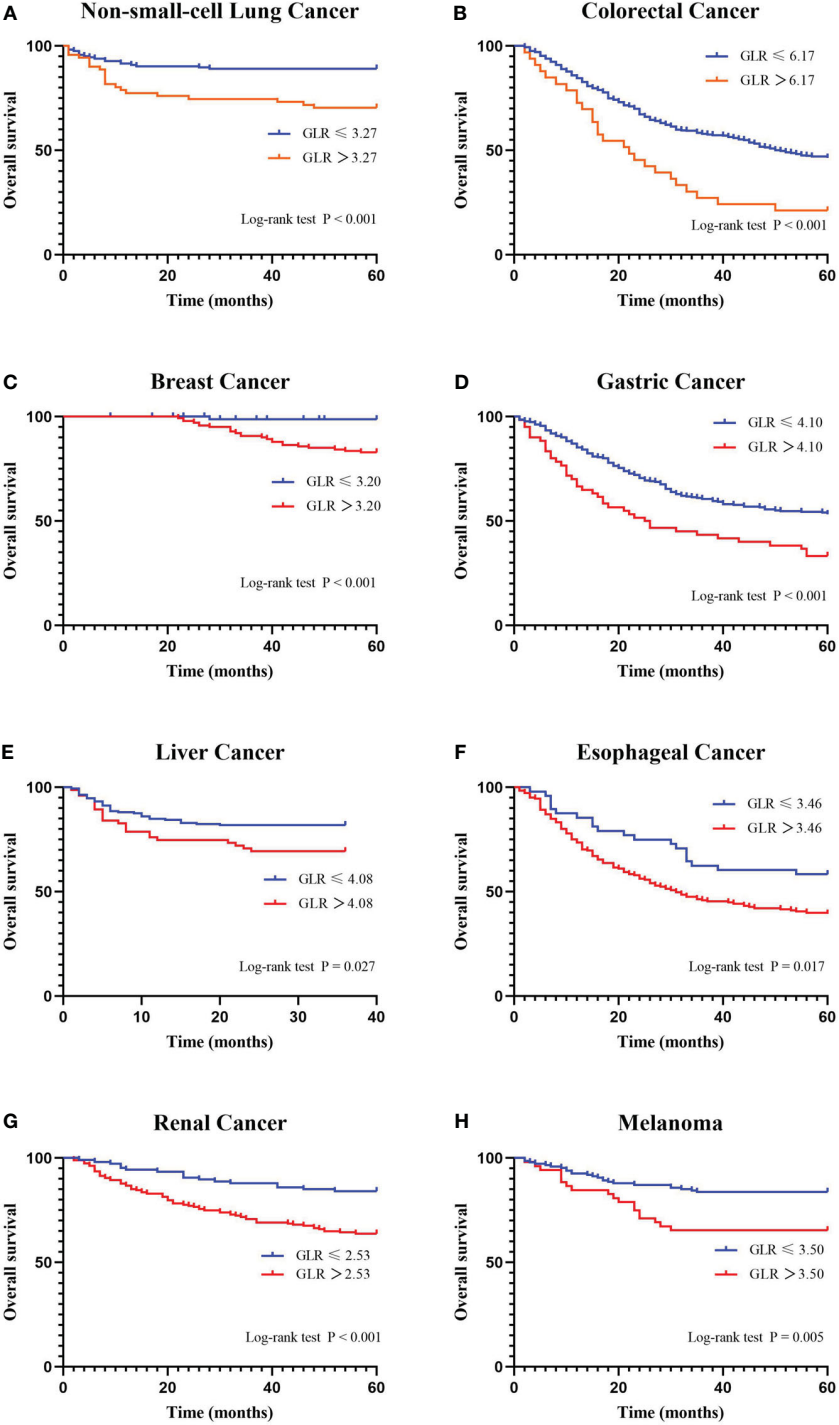
Kaplan-Meier curves for overall survival stratifed by preoperative GLR. Overall survival Kaplan-Meier survival curves according to GLR levels for patients who underwent radical surgery. The 5-year overall survival in patients with high GLR or low GLR is plotted. Kaplan-Meier analysis demonstrated that high GLR was significantly associated with the shorter overall survival. **(A)** non-small-cell lung cancer; **(B)** colorectal cancer; **(C)** breast cancer; **(D)** gastric cancer; **(E)** liver cancer; **(F)** esophageal cancer; **(G)** renal cancer; and **(H)** melanoma. GLR, glucose to lymphocyte ratio.

A ROC curve analysis was constructed to determine the optimal cutoff value for GLR in different tumor types (**Figure 1**). Based on the analysis of receiver operating characteristic curves, the optimal GLR cut-off values for gastric, renal, colorectal, non-small cell lung, breast, liver, esophageal, and melanoma cancers were 4.1, 2.53, 6.17, 3.27, 3.2, 4.08, 3.46, and 3.5, respectively. And the corresponding sensitivity and specificity are shown in **Figure 1**. Patients were classified as having high or low preoperative GLR according to cut-off values. We found that elevated GLR significantly predicted overall survival (**Figure 2**). Among patients with non-small cell lung cancer, 72 (30%) had higher preoperative GLR levels. With a median follow-up of 60 months, 43 (17.9%) patients had death events. 22 patients with GLR > 3.27 and 21 patients with GLR ≤ 3.27 had death events. Overall survival was significantly shorter in patients with high GLR (n=72) versus those with low GLR (n=168) (p < 0.001). The mean survival time was 45.5 months for patients with GLR > 3.27 and 53.4 months for patients with GLR ≤ 3.27, respectively. Kaplan-Meier OS curves for normal versus increased GLR showed a notable separation (**Figure 2A**). In patients with colorectal cancer, there were 212 (56.1%) patients who had death events. Compared to those with low GLR levels, the patients with high GLR levels had significantly shorter overall survival (survival rates of 21.2% and 46.1%, respectively, p < 0.001; **Figure 2B**). In breast cancer, OS was lower in high-GLR subjects than in low-GLR counterparts (mean survival time, 54.1 months vs 55.9 months, p < 0.001; **Figure 2C**). In gastric cancer, the OS rate was markedly worse in the high-GLR group than that in the low-GLR group (5-year survival rates of 32.3% and 53.1%, respectively, p < 0.001; **Figure 2D**). In liver cancer, OS was lower in high-GLR subjects than that in low-GLR counterparts (mean survival time, 27.3 months vs 30.6 months, p = 0.027; **Figure 2E**). Among patients with renal cancer, the high GLR grade group had a worse OS than the low GLR grade group (mean survival time, 46.1 months vs 54.3 months, p < 0.001; **Figure 2G**). Similarly, in melanoma, subjects with a high GLR have a shorter OS compared to patients with a lower GLR (mean survival time, 44.9 months vs 52.8 months, p = 0.005; **Figure 2H**). And in esophageal cancer, OS was lower in high-GLR subjects than in low-GLR subjects (mean survival time, 34.7 months vs 43.9 months, p = 0.017; **Figure 2F**).

The univariate and multivariate analyses were performed to evaluate the preoperative predictors for OS (**Table 2**). According to the univariate analysis, GLR, gender, adjuvant chemotherapy, histology, clinical stage, and white blood cell were significantly correlated with OS in patients with NSCLC. In colorectal cancer, GLR, age, T classification, lymph node status, clinical stage, hemoglobin, and white blood cell were related to OS. In gastric cancer, GLR, age, tumor size, histology, T classification, lymph node status, clinical stage, carcinoma embryonic antigen (CEA), BMI, and white blood cell were in correlation with OS. In patients with renal cancer, GLR, age, hypertension, diabetes mellitus, tumor size, T classification, lymph node status, clinical stage, hemoglobin, and platelet count were significantly related to OS. In melanoma, GLR, lymph node status, and clinical stage were prognostic‐related risk factors for OS. In patients with liver cancer, GLR, hypertension, tumor size, T classification, lymph node status, clinical stage, and white blood cell were related to OS. In esophageal cancer, GLR, T classification, lymph node status, and clinical stage were significantly related to OS. And, in breast cancer, GLR, progesterone receptor (PR), human epidermal growth factor receptor-2 (HER-2), Ki-67, T classification, lymph node status, clinical stage, hypertension, BMI, and platelet count were significantly related to OS. Next, the variables showing statistical significance in the univariate analysis (p < 0.10) were included in the multivariate analysis. In multivariate analysis, GLR was identified as an independent prognostic factor for OS in different tumor types.

**Table 2:** Univariate analysis and Multivariate analysis of overall survival in cancer patients.

Variables	Univariate Analysis		Multivariate Analysis	
	Hazard ratio (95% CI)	*P*	Hazard ratio (95% CI)	*P*
Non-small-cell Lung Cancer				
GLR	1.477 (1.164 - 1.875)	0.001	1.602 (1.245 - 2.061)	**< 0.001**
Age (years)	1.025 (0.995 - 1.057)	0.109		
Gender (Male vs Female)	2.365 (1.134 - 4.931)	0.022	2.883 (1.280 - 6.491)	**0.011**
Hypertension (Yes vs No)	1.061 (0.472 - 2.385)	0.885		
Diabetes mellitus (Yes vs No)	1.013 (0.313 - 3.276)	0.982		
Tumor size (≥ 4cm vs < 4cm)	1.084 (0.573 - 2.051)	0.805		
Smoking history (Yes vs No)	1.473 (0.809 - 2.682)	0.206		
Adjuvant chemotherapy (Yes vs No)	0.287 (0.133 - 0.618)	0.001	0.454 (0.183 - 1.126)	0.088
Histology (Others vs Adenocarcinoma)	1.834 (1.001 - 3.362)	0.050	1.246 (0.659 - 2.357)	0.499
T classification (T3/T4 vs T1/T2)	1.092 (0.430 - 2.774)	0.853		
Lymph node status (Present vs Absent)	1.065 (0.569 - 1.994)	0.843		
Clinical Stage (III vs I/II)	2.778 (1.525 - 5.060)	0.001	1.502 (0.984 - 2.292)	0.059
BMI (kg/m^2^)	1.025 (0.930 - 1.129)	0.623		
WBC (×10^9^/L)	1.161 (1.069 - 1.262)	< 0.001	1.170 (1.068 - 1.281)	**0.001**
Hemoglobin (g/dl)	0.994 (0.980 - 1.009)	0.461		
Platelet count (×10^9^/L)	1.003 (0.999 - 1.007)	0.156		
Colorectal Cancer				
GLR	1.073 (1.029 - 1.120)	0.001	1.051 (1.005 - 1.100)	**0.030**
Age (years)	1.019 (1.003 - 1.034)	0.016	1.021 (1.006 - 1.037)	**0.006**
Gender (Male vs Female)	1.062 (0.801 - 1.408)	0.675		
Hypertension (Yes vs No)	0.775 (0.562 - 1.069)	0.121		
Diabetes mellitus (Yes vs No)	1.146 (0.775 - 1.696)	0.495		
T classification (T3/T4 vs T1/T2)	2.884 (1.799 - 4.625)	< 0.001	1.840 (1.124 - 3.012)	**0.015**
Lymph node status (Present vs Absent)	2.591 (1.963 - 3.418)	< 0.001	0.719 (0.420 - 1.230)	0.228
Clinical Stage (III/IV vs I/II)	3.129 (2.342 - 4.181)	< 0.001	3.764 (2.125 - 6.667)	**< 0.001**
BMI/m (kg^2^)	0.983 (0.941 - 1.027)	0.446		
WBC (×10^9^/L)	1.097 (1.037 - 1.161)	0.001	1.090 (1.031 - 1.152)	**0.002**
Hemoglobin (g/dl)	0.994 (0.989 - 0.999)	0.032	0.995 (0.990 - 1.001)	0.082
Platelet count (×10^9^/L)	1.000 (0.998 - 1.001)	0.722		
Breast Cancer				
GLR	14.693 (1.988 - 108.615)	0.008	13.015 (1.683 - 100.676)	**0.014**
Age (years)	1.027 (0.988 - 1.068)	0.176		
Hypertension (Yes vs No)	2.710 (1.132 - 6.489)	0.025	0.578 (0.187 - 1.787)	0.341
Diabetes mellitus (Yes vs No)	0.047 (0.000 - 711.277)	0.534		
Menopausal status (Post vs Pre)	0.800 (0.363 - 1.762)	0.579		
ER (Positive vs Negative)	0.663 (0.301 - 1.459)	0.307		
PR (Positive vs Negative)	2.362 (0.886 - 6.293)	0.086	1.578 (0.531 - 4.684)	0.412
HER2 status (Positive vs Negative)	3.527 (1.323 - 9.399)	0.012	1.879 (0.637 - 5.547)	0.253
Ki-67 (≥ 20% vs < 20%)	4.656 (1.944 - 11.152)	0.001	2.118 (0.697 - 6.436)	0.186
Tumor size (≥ 2.5cm vs < 2.5cm)	1.827 (0.788 - 4.235)	0.160		
T classification (T3/T4 vs T1/T2)	4.306 (1.285 - 14.437)	0.018	1.041 (0.213 - 5.086)	0.961
Lymph node status (Present vs Absent)	42.730 (14.571 - 125.306)	< 0.001	24.641 (5.956 - 101.939)	**< 0.001**
Clinical stage(III vs I/II)	21.082 (9.358 - 47.496)	< 0.001	1.401 (0.482 - 4.066)	0.536
BMI (kg/m^2^)	1.094 (1.000 - 1.196)	0.050	1.041 (0.906 - 1.195)	0.570
WBC (×10^9^/L)	1.069 (0.874 - 1.308)	0.516		
Hemoglobin (g/dl)	1.007 (0.974 - 1.041)	0.665		
Platelet count (×10^9^/L)	1.009 (1.002 - 1.016)	0.015	1.005 (0.995 - 1.014)	0.331
Gastric Cancer				
GLR	1.201 (1.082 - 1.334)	0.001	1.169 (1.055 - 1.295)	**0.003**
Age (years)	1.024 (1.008 - 1.040)	0.002	1.025 (1.009 - 1.041)	**0.003**
Gender (Male vs Female)	1.012 (0.728 - 1.408)	0.942		
Hypertension (Yes vs No)	1.037 (0.699 - 1.538)	0.858		
Diabetes mellitus (Yes vs No)	1.211 (0.688 - 2.133)	0.506		
Tumor size (> 5cm vs ≤ 5cm)	1.390 (1.022 - 1.892)	0.036	1.055 (0.764 - 1.456)	0.745
Histology (Poor vs Well/Moderate)	1.681 (1.083 - 2.609)	0.021	1.673 (1.051 - 2.662)	**0.030**
T classification (T3/T4 vs T1/T2)	1.824 (1.215 - 2.737)	0.004	1.409 (0.888 - 2.236)	0.145
Lymph node status (Present vs Absent)	2.904 (1.781 - 4.736)	< 0.001	1.767 (0.980 - 3.188)	0.058
Clinical Stage (III/IV vs I/II)	2.265 (1.601 - 3.204)	< 0.001	1.326 (0.839 - 2.095)	0.227
CEA (> 5 ng/mL vs ≤ 5 ng/mL)	1.791 (1.260 - 2.547)	0.001	1.315 (0.898 - 1.925)	0.159
BMI (kg/m^2^)	0.938 (0.896 - 0.982)	0.007	0.922 (0.878 - 0.969)	**0.001**
WBC (×10^9^/L)	1.140 (1.066 - 1.219)	< 0.001	1.136 (1.060 - 1.216)	**< 0.001**
Hemoglobin (g/dl)	0.998 (0.992 - 1.003)	0.390		
Platelet count (×10^9^/L)	1.001 (0.999 - 1.002)	0.200		
Liver Cancer				
GLR	1.809 (1.079 - 3.033)	0.024	2.233 (1.277 - 3.904)	**0.005**
Age (years)	1.014 (0.986 - 1.043)	0.321		
Gender (Male vs Female)	0.861 (0.518 - 1.432)	0.564		
Tumor size (≥ 5 cm vs< 5 cm)	2.811 (1.586 - 4.984)	< 0.001	1.924 (0.945 - 3.916)	0.071
Smoker (Yes vs No)	1.085 (0.645 - 1.826)	0.758		
Drinking (Yes vs No)	0.844 (0.415 - 1.714)	0.639		
Hypertension (Yes vs No)	2.211 (1.196 - 4.088)	0.011	1.723 (0.909 - 3.267)	0.095
Hepatitis B (Present vs Absent)	0.692 (0.391 - 1.227)	0.207		
Liver Cirrhosis (Present vs Absent)	0.728 (0.437 - 1.213)	0.222		
T classification (T3/T4 vs T1/T2)	2.244 (1.338 - 3.762)	0.002	0.703 (0.187 - 2.641)	0.601
Lymph node status (Present vs Absent)	3.843 (1.945 - 7.595)	< 0.001	2.087 (0.832 - 5.234)	0.117
Clinical Stage (III/IV vs I/II)	2.725 (1.592 - 4.663)	< 0.001	1.984 (0.486 - 8.105)	0.340
BMI (kg/m^2^)	1.021 (0.939 - 1.110)	0.629		
WBC (×10^9^/L)	1.182 (1.045 - 1.337)	0.008	1.153 (1.007 - 1.320)	**0.040**
Hemoglobin (g/dl)	1.003 (0.989 - 1.018)	0.661		
Platelet count (×10^9^/L)	1.002 (0.998 - 1.005)	0.323		
Esophageal Cancer				
GLR	1.771 (1.100 - 2.852)	0.019	1.925 (1.190 - 3.114)	**0.008**
Age (years)	1.006 (0.983 - 1.029)	0.624		
Gender (Male vs Female)	0.673 (0.353 - 1.284)	0.230		
Tumor size (≥ 3.5 cm vs < 3.5 cm)	1.266 (0.794 - 2.019)	0.321		
Smoker (Yes vs No)	0.774 (0.513 - 1.169)	0.224		
Drinking (Yes vs No)	0.705 (0.442 - 1.125)	0.143		
Hypertension (Yes vs No)	0.647 (0.371 - 1.126)	0.123		
Diabetes mellitus (Yes vs No)	1.437 (0.671 - 3.079)	0.351		
Histology (Others vs Squamous carcinoma)	1.564 (0.639 - 3.824)	0.327		
T classification (T3/T4 vs T1/T2)	1.707 (1.202 - 2.424)	0.003	1.418 (0.567 - 3.545)	0.455
Lymph node status (Present vs Absent)	1.980 (1.399 - 2.802)	< 0.001	1.778 (1.238 - 2.553)	**0.002**
Clinical Stage (III/IV vs I/II)	1.794 (1.253 - 2.568)	0.001	1.184 (0.459 - 3.058)	0.727
BMI (kg/m^2^)	0.981 (0.927 - 1.039)	0.512		
WBC (×10^9^/L)	1.022 (0.940 - 1.111)	0.608		
Hemoglobin (g/dl)	0.995 (0.983 - 1.007)	0.408		
Platelet count (×10^9^/L)	1.001 (0.999 - 1.004)	0.273		
Renal Cancer				
GLR	1.153 (1.068 - 1.245)	< 0.001	1.139 (1.054 - 1.232)	**0.001**
Age (years)	1.017 (0.997 - 1.037)	0.098	1.006 (0.984 - 1.028)	0.620
Gender (Male vs Female)	1.430 (0.891 - 2.293)	0.138		
Smoker (Yes vs No)	1.363 (0.755 - 2.460)	0.304		
Hypertension (Yes vs No)	1.728 (1.055 - 2.829)	0.030	1.262 (0.730 - 2.180)	0.405
Diabetes mellitus (Yes vs No)	2.373 (1.358 - 4.148)	0.002	1.518 (0.773 - 2.980)	0.226
Drinking (Yes vs No)	0.813 (0.298 - 2.219)	0.686		
Tumor size (> 4 cm vs ≤ 4 cm)	2.360 (1.441 - 3.866)	0.001	1.779 (1.054 - 3.003)	**0.031**
Histology (Clear cell vs Others)	2.226 (0.816 - 6.074)	0.118		
T classification (T3/T4 vs T1/T2)	4.606 (2.622 - 8.093)	< 0.001	0.811 (0.364 - 1.807)	0.608
Lymph node status (Present vs Absent)	4.738 (2.771 - 8.100)	< 0.001	2.018 (1.010 - 4.034)	**0.047**
Clinical Stage (III/IV vs I/II)	5.169 (3.287 - 8.130)	< 0.001	3.463 (1.815 - 6.606)	**< 0.001**
BMI (kg/m^2^)	0.963 (0.902 - 1.029)	0.265		
WBC (×10^9^/L)	1.058 (0.966 - 1.158)	0.226		
Hemoglobin (g/dl)	0.977 (0.968 - 0.987)	< 0.001	0.998 (0.984 - 1.012)	0.761
Platelet count (×10^9^/L)	1.005 (1.003 - 1.007)	< 0.001	1.005 (1.002 - 1.007)	**0.001**
Melanoma				
GLR	1.519 (1.172 - 1.968)	0.002	1.486 (1.120 - 1.972)	**0.006**
Age (years)	0.990 (0.967 - 1.013)	0.388		
Gender (Male vs Female)	0.787 (0.430 - 1.443)	0.439		
Hypertension (Yes vs No)	1.538 (0.648 - 3.651)	0.329		
Diabetes mellitus (Yes vs No)	1.342 (0.324 - 5.553)	0.685		
Tumor location (Sun-exposed vs Sun-protected)	1.245 (0.489 - 3.169)	0.645		
Ulceration (Yes vs No)	1.413 (0.735 - 2.718)	0.300		
Histology (SSM/NM vs ALM/LMM/others)	0.653 (0.354 - 1.203)	0.172		
T classification (T3/T4 vs T1/T2)	1.236 (0.572 - 2.670)	0.590		
Lymph node status (Present vs Absent)	2.957 (1.613 - 5.421)	< 0.001	1.054 (0.347 - 3.196)	0.926
Clinical stage (III/IV vs I/II)	3.582 (1.943 - 6.604)	< 0.001	3.228 (1.057 - 9.859)	**0.040**
BMI (kg/m^2^)	0.933 (0.850 - 1.023)	0.140		
WBC (×10^9^/L)	0.868 (0.725 - 1.040)	0.125		
Hemoglobin (g/dl)	0.996 (0.983 - 1.010)	0.574		
Platelet count (×10^9^/L)	1.001 (0.996 - 1.005)	0.719		

Supplement: SSM, superficial spreading melanoma; NM, nodular melanoma; ALM, acromacular melanoma; LMM, lentigo maligna melanoma.

Bold values mean P < 0.05.

## Discussion

In this study, we retrospectively analyzed the predictive value of preoperative GLR in patients with CRC, NSCLC, GC, EC, BC, renal cancer, liver cancer, and melanoma. It was found that increased GLR was markedly associated with shorter OS.

Previous studies have proven that GLR is a prognostic marker for some tumors, such as CRC (14), pancreatic carcinoma (12) and PT2 gallbladder carcinoma (15). Our study was consistent with the above results. In addition, our results showed the prognostic value of preoperative GLR in other cancers. Consistent with previous studies (16–19), our findings confirmed that age, BMI, WBC, and platelet count were independently associated with OS in the multivariate analysis in some cancers.

GLR is derived from the ratio of blood glucose to lymphocyte count (20). Altered glucose metabolism is a marked trait of cancer. Therefore, it is worth considering that tumor cell glycolytic activity increases when blood glucose is elevated, and then cancer cells transport extracellular glucose through the cytoplasm, leading to an increase in intracellular glucose, whose fermentation into lactic acid generates energy that activates cellular signaling pathways, thereby mediating the spread, invasion, and metastasis of cancer cells (21). It has been confirmed that the diabetes caused by hyperglycemia gives rise to hyperinsulinemia and insulin resistance, which may lead to changes in the tumor microenvironment by producing irreversible glycation end products or by affecting the expression of angiogenic factors and the acidity of the microenvironment, promoting tumor development, and even increasing tumor metastasis and resistance to chemotherapy (22–24). Also, the abysmal outcome of hyperglycemia is associated with chronic subclinical inflammation, referred to as “meta-inflammation”. Chronic subclinical inflammation exacerbates hyperglycemia by modulating insulin resistance, leading to a series of diabetic complications, while hyperglycemia promotes the production of free radicals, leading to inflammation and metabolic disorders, thus creating a vicious cycle that exacerbates disease progression (25, 26). These form the basis of a poorer prognosis for tumor patients. Moreover, lymphocytes have an essential role in immune regulation and the prevention of tumor development. On the one hand, lymphocytes suppress cancer progression by inhibiting cell proliferation and promoting cell death (24). Several reports have revealed that lymphocytes can activate a cell-mediated immune response and stimulate the release of cytokines such as interferon and TNF-α to exert organismal protective effects, even leading to the lysis of tumor cells (27–29). On the other hand, cumulative evidence demonstrated that lymphocytes could indicate the nutritional status of patients (30). In brief, elevated GLR, that is, high glucose and low lymphocyte count, is strongly associated with cancer progression and worse OS, which is in accordance with our findings.

Compared with the existing studies, this research involved a wide range of diseases, and the results were more comprehensive. However, our research had some limitations. Firstly, the study has a retrospective design and the sample size was not large enough. Secondly, the potential confounders that may exist (e.g., drug administration, patient selection, and surgical procedures) may have caused the sampling error. Thirdly, the cut-off values for specific cancer types are required for further evaluation in the future. Finally, further investigation is needed regarding the mechanisms at the molecular level. Moreover, serum lactate and inflammatory cytokines, such as TNFα or IL-10, should be detected in future studies.

GLR is a simple, cost-effective, and noninvasive parameter in clinical practice. Our study revealed the prognostic value of preoperative GLR in some resectable tumors. Future prospective studies are required to confirm the findings. Moreover, it would be interesting to investigate whether adding GLR to other prognosis scores could improve their performance.

In conclusion, elevated preoperative GLR was remarkably associated with a poorer prognosis in patients with NSCLC, CRC, breast cancer, gastric cancer, kidney cancer, liver cancer, esophageal cancer, and melanoma. Preoperative GLR promises to be an essential predictor of survival for cancer patients.

## Data availability statement

The raw data supporting the conclusions of this article will be made available by the authors, without undue reservation.

## Ethics statement

This research was in strict compliance with the Helsinki Declaration. This study was approved by our Institutional Review Board (approval number KY2022-10). As the study was retrospective, written informed consent was waived.

## Author contributions

LL: Data curation, Writing – original draft. BZ: Investigation, Writing – original draft, Data analysis. RW: Formal analysis, Methodology, Writing – review & editing. WH: Data curation, Investigation, Software, Writing – review & editing. YN: Conceptualization, Supervision, Validation, Writing – review & editing. WW: Investigation, Validation, Visualization, Writing – review & editing. QJ: Data curation, Methodology, Supervision, Writing – original draft. JY: Formal analysis, Validation, Visualization, Writing – review & editing. GW: Conceptualization, Methodology, Writing – review & editing. SM: Software, Supervision, Validation, Writing – review & editing. YL: Formal analysis, Investigation, Visualization, Writing – review & editing.

## References

[1] Siegel RL Miller KD Fuchs HE Jemal A. Cancer statistics, 2022. CA Cancer J Clin. (2022) 7:33. doi: 10.3322/caac.21708. 35020204

[2] Wyld L Audisio RA Poston GJ. The evolution of cancer surgery and future perspectives. Nat Rev Clin Oncol. (2015) 115:124. doi: 10.1038/nrclinonc.2014.191. 25384943

[3] Magliano DJ Sacre JW Harding JL Gregg EW Zimmet PZ Shaw JE. Young-onset type 2 diabetes mellitus - implications for morbidity and mortality. Nat Rev Endocrinol. (2020) 321:331. doi: 10.1038/s41574-020-0334-z. 32203408

[4] Barone BB Yeh HC Snyder CF Peairs KS Stein KB Derr RL. Long-term all-cause mortality in cancer patients with preexisting diabetes mellitus: a systematic review and meta-analysis. JAMA. (2008) 2754:2764. doi: 10.1001/jama.2008.824. PMC3093051 19088353

[5] Suh S Kim KW. Diabetes and cancer: cancer should be screened in routine diabetes assessment. Diabetes Metab J. (2019) 733:743. doi: 10.4093/dmj.2019.0177. PMC6943263 31902143

[6] Tsilidis KK Kasimis JC Lopez DS Ntzani EE Ioannidis JP. Type 2 diabetes and cancer: umbrella review of meta-analyses of observational studies. BMJ. (2015) 350:g7607. doi: 10.1136/bmj.g7607. 25555821

[7] Bánhegyi RJ Gazdag A Rácz B Beke S Fülöp N Onkodiabetológia I. Metabolikus és molekuláris összefüggések a rosszindulatú daganatok és a cukorbetegség között [Oncodiabetology I. Metabolic and molecular relationships between cancer and diabetes]. Orv Hetil. (2022) 163(39):1535–43. doi: 10.1556/650.2022.32564 36153724

[8] Zhang K Bai P Dai H Deng Z. Metformin and risk of cancer among patients with type 2 diabetes mellitus: A systematic review and meta-analysis. Prim Care Diabetes. (2021) 52:58. doi: 10.1016/j.pcd.2020.06.001. 32605879

[9] Hwangbo Y Kang D Kang M Kim S Lee EK Kim YA. Incidence of diabetes after cancer development: A korean national cohort study. JAMA Oncol. (2018) 1099:1105. doi: 10.1001/jamaoncol.2018.1684. PMC6143049 29879271

[10] Paijens ST Vledder A De Bruyn M Nijman HW. Tumor-infiltrating lymphocytes in the immunotherapy era. Cell Mol Immunol. (2021) 842:859. doi: 10.1038/s41423-020-00565-9. PMC8115290 33139907

[11] Wu SY Fu T Jiang YZ Shao ZW. Natural killer cells in cancer biology and therapy. Mol Cancer. (2020) 19(1):120. doi: 10.1186/s12943-020-01238-x. 32762681 PMC7409673

[12] Zhong A Cheng CS Kai J Lu R Guo L. Clinical significance of glucose to lymphocyte ratio (GLR) as a prognostic marker for patients with pancreatic cancer. Front Oncol. (2020) 520330. doi: 10.3389/fonc.2020.520330. PMC7561421 33117673

[13] McShane LM Altman DG Sauerbrei W Taube SE Gion M Clark GM. Reporting recommendations for tumor marker prognostic studies (REMARK). J Natl Cancer Inst. (2005) 1180:1184. doi: 10.1093/jnci/dji237. 16106022

[14] Yang M Zhang Q Ge Y Tang M Zhang X Song M. Glucose to lymphocyte ratio predicts prognoses in patients with colorectal cancer [published online ahead of print, 2022 Dec 7]. Asia Pac J Clin Oncol. (2022) 19(4):542–8. doi: 10.1111/ajco.13904. 36479824

[15] Navarro J Kang I Hwang HK Yoon DS Lee WJ Kang CW. Glucose to lymphocyte ratio as a prognostic marker in patients with resected pT2 gallbladder cancer. J Surg Res. (2019) 17:29. doi: 10.1016/j.jss.2019.02.043. 30909062

[16] Laconi E Marongiu F Degregori J. Cancer as a disease of old age: changing mutational and microenvironmental landscapes. Br J Cancer. (2020) 943:52. doi: 10.1038/s41416-019-0721-1. PMC7109142 32042067

[17] Schwartz SS Grant S FA Herman ME. Intersections and clinical translations of diabetes mellitus with cancer promotion, progression and prognosis. Postgrad Med. (2019) 597:606. doi: 10.1080/00325481.2019.1657358. 31419922

[18] Smeda M Przyborowski K Stojak M Chlopicki S. The endothelial barrier and cancer metastasis: Does the protective facet of platelet function matter? Biochem Pharmacol. (2020) 176:113886. doi: 10.1016/j.bcp.2020.113886. 32113813

[19] Xu H Zheng X Ai J Yang L. Hemoglobin, albumin, lymphocyte, and platelet (HALP) score and cancer prognosis: A systematic review and meta-analysis of 13,110 patients. Int Immunopharmacol. (2023) 114:109496. doi: 10.1016/j.intimp.2022.109496. 36462339

[20] Li L Zou G Liu J. Preoperative glucose-to-lymphocyte ratio is an independent predictor for acute kidney injury after cardiac surgery in patients in intensive care unit. Int J Gen Med. (2021) 6529:6537. doi: 10.2147/IJGM.S335896. PMC8518472 34675620

[21] Wahdan-Alaswad R Fan Z Edgerton SM Liu B Deng XS Arnadottir SS. Glucose promotes breast cancer aggression and reduces metformin efficacy. Cell Cycle. (2013) 3759:3769. doi: 10.4161/cc.26641. PMC3905068 24107633

[22] Chott A Sun Z Morganstern D Pan J Li T Susani M. Tyrosine kinases expressed *in vivo* by human prostate cancer bone marrow metastases and loss of the type 1 insulin-like growth factor receptor. Am J Pathol. (1999) 1271:1279. doi: 10.1016/S0002-9440(10)65229-7. PMC1867033 10514409

[23] Li W Zhang X Sang H Zhou Y Shang C Wang Y. Effects of hyperglycemia on the progression of tumor diseases. J Exp Clin Cancer Res. (2019) 38(1):327. doi: 10.1186/s13046-019-1309-6. 31337431 PMC6651927

[24] Siska PJ Rathmell JC. T cell metabolic fitness in antitumor immunity. Trends Immunol. (2015) 257:264. doi: 10.1016/j.it.2015.02.007. PMC4393792 25773310

[25] Berbudi A Rahmadika N Tjahjadi AI Ruslami R. Type 2 diabetes and its impact on the immune system. Curr Diabetes Rev. (2020) 442:449. doi: 10.2174/1573399815666191024085838. PMC7475801 31657690

[26] De Heredia FP Gómez-Martínez S Marcos A. Obesity, inflammation and the immune system. Proc Nutr Soc. (2012) 332:338. doi: 10.1017/S0029665112000092. 22429824

[27] Greten FP Grivennikov SI. Inflammation and cancer: triggers, mechanisms, and consequences. Immunity. (2019) 27:41. doi: 10.1016/j.immuni.2019.06.025. PMC6831096 31315034

[28] Koliaraki V Prados A Armaka M Kollias G. The mesenchymal context in inflammation, immunity and cancer. Nat Immunol. (2020) 974:982. doi: 10.1038/s41590-020-0741-2. 32747813

[29] Lin DZ Qu N Shi RL Lu ZW Ji QH Wu WL. Risk prediction and clinical model building for lymph node metastasis in papillary thyroid microcarcinoma. Onco Targets Ther. (2016) 5307:5316. doi: 10.2147/OTT. PMC5004998 27601922

[30] Reljic D Herrmann HJ Neurath MF Zopf Y. Iron Beats Electricity: Resistance Training but Not Whole-Body Electromyostimulation Improves Cardiometabolic Health in Obese Metabolic Syndrome Patients during Caloric Restriction-A Randomized-Controlled Study. Nutrients. (2021) 13(5):1604. doi: 10.3390/nu13051640. 34068089 PMC8152778

